# Silent Synergy: Is the Presence of Concurrent β-Lactamase Mechanisms Undermining Antimicrobial Effectiveness?

**DOI:** 10.7759/cureus.109670

**Published:** 2026-05-26

**Authors:** Shanu Sharma, Geeta Karande, Satish Patil

**Affiliations:** 1 Department of Microbiology, Krishna Institute of Medical Sciences, Krishna Vishwa Vidyapeeth (Deemed to be University), Karad, IND

**Keywords:** antimicrobial resistance, clsi, concurrent β-lactamases, enterobacteriaceae, infection control

## Abstract

Background

The worldwide escalation of antimicrobial resistance poses a substantial challenge to the effective management of both community-acquired and hospital-associated infections. Among Gram-negative bacteria, resistance to β-lactam antibiotics is predominantly mediated by enzymes such as extended-spectrum β-lactamases, AmpC β-lactamases, and metallo-β-lactamases.

Aim

This study aimed to identify the concurrent production of these β-lactamase mechanisms in Gram-negative clinical isolates.

Materials and methods

A cross-sectional laboratory-based study was performed on 185 non-duplicate Enterobacteriaceae isolates obtained from clinical specimens. All the isolates underwent phenotypic screening for extended spectrum of β-lactamases (ESBL), AmpC, and metallo-β-lactamases (MBL) production using standard disk diffusion-based methods. The study adhered to ethical guidelines with institutional approval. Data were analyzed statistically using the Chi-square test, with significance set at p < 0.05.

Results

Out of 185 Enterobacteriaceae isolates, *Klebsiella pneumoniae* (90; 50.0%) and *Escherichia coli* (60; 33.3%) were the most prevalent. The maximum number of isolates was observed in males aged 41-60 years and females aged 21-40 years. Of 145 MDR isolates, 63 (43.4%) co-expressed two or three β-lactamases, with 28 (19.3%) showing MBL + ESBL, 13 (8.9%) MBL + AmpC, 16 (11%) ESBL + AmpC, and 6 (4.1%) concurrently producing MBL, ESBL, and AmpC.

Conclusion

Over the course of the study, this investigation demonstrated the substantial burden of multidrug-resistant Enterobacteriaceae, with 63 (43.4%) isolates co-expressing multiple β-lactamases and 6 (4.1%) simultaneously producing MBL, ESBL, and AmpC, highlighting a critical challenge. Incorporating routine screening for these enzymes is essential to manage infection and limit the spread of resistance.

## Introduction

The members of the Enterobacteriaceae family are Gram-negative rods, characterized by facultative anaerobiosis and versatile metabolic activity. They constitute a core component of the gastrointestinal microbiota in humans and animals, persisting predominantly as commensal organisms [1]. Although typically benign within the intestinal ecosystem, members are found to exhibit considerable taxonomic diversity, encompassing numerous species of substantial clinical significance. The progressive advent and dissemination of multidrug-resistant Gram-negative bacteria over the past decades have intensified, constituting a critical challenge to healthcare systems worldwide [2]. Recent analyses have documented the simultaneous expression of multiple β-lactamase enzymes (metallo-β-lactamases, extended-spectrum β-lactamases, and AmpC β-lactamases) within individual bacterial isolates a phenomenon that amplifies antimicrobial resistance and presents pronounced obstacles to optimal therapeutic intervention [3]. Although carbapenems serve as the first-line resort to combat infections caused by multidrug-resistant strains, the traditional assumption of universal carbapenem susceptibility among members of this family is no longer valid [4]. These observations illuminate the exigent necessity for microbiologists to delineate β-lactamase-producing Enterobacteriaceae. Data procured through such methodical evaluations are of paramount significance in guiding evidence-based antimicrobial therapy and in the continual refinement of therapeutic protocols. Building on the preceding rationale, the research aims to investigate the prevalence of multidrug-resistant Enterobacteriaceae and to characterize the concurrent expression of multiple β-lactamases, including metallo-β-lactamases (MBL), extended spectrum of β-lactamases (ESBLs), and AmpC in clinical isolates.

## Materials and methods

A cross-sectional laboratory-based study was carried out in the Department of Microbiology, Krishna Institute of Medical Sciences (KIMS), Karad. A total of 185 non-repetitive Enterobacteriaceae isolates, obtained from diverse specimens, were collected with representation from both male and female patients. Isolates from the same patient or duplicate specimens were excluded to prevent redundancy. The study was conducted in compliance with ethical principles governing biomedical research involving human participants and received prior approval from the Institutional Ethics Committee (068/2021-22). All laboratory investigations were performed using standard microbiological techniques in accordance with standard operating procedure [5].

**Figure 1:**
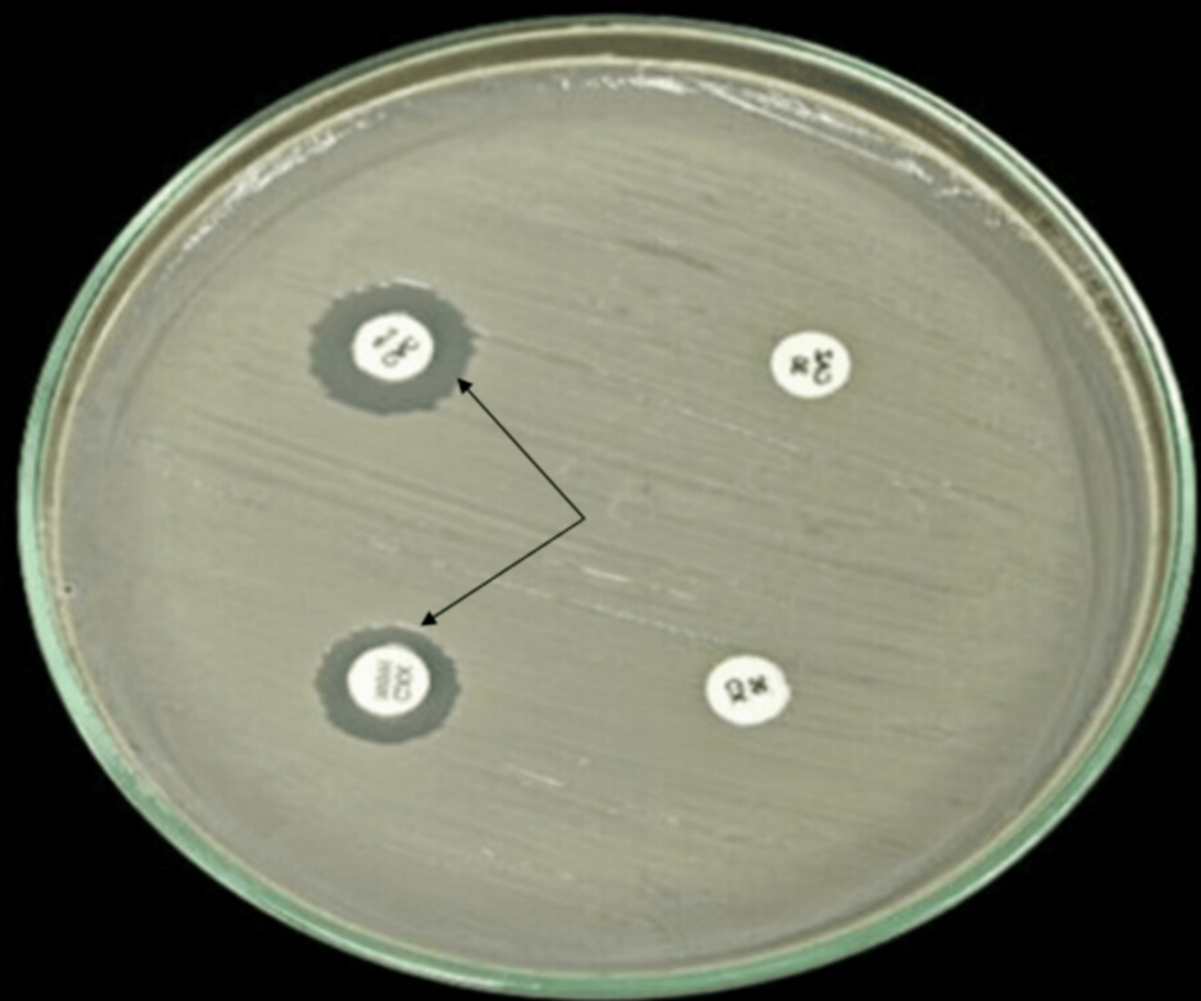
ESBL + AmpC production ESBL: extended spectrum of β-lactamases; AmpC: AmpC β-lactamases. The arrows indicate the coproduction of ESBL and AmpC β-lactamases in the test isolate.

The assessment of MBL, ESBL, and AmpC β-lactamase was conducted using disk diffusion methods. MBL production was confirmed with the Imipenem-ethylenediaminetetraacetic acid (EDTA) combined disk test, where an increase of ≥7 mm in the inhibition zone around the imipenem + EDTA disc compared to imipenem alone indicated MBL activity [6]. ESBL production was identified using the combined disk diffusion test (CDDT) by measuring a ≥5 mm increase in the inhibition zone around ceftazidime + clavulanic acid discs compared to ceftazidime alone [7]. AmpC production was assessed using the phenylboronic acid disc test, where a ≥5 mm increase in the inhibition zone around cefoxitin + phenylboronic acid discs, compared to cefoxitin alone, indicated AmpC activity [8]. All the test procedures were executed as per the standard protocols with incubation at 37°C for 20-24 hours. The data so obtained were systematically analyzed using GraphPad InStat software (GraphPad Software, San Diego, California, USA), and their significance was assessed using the Chi-square test. A p-value of less than 0.05 was considered indicative of a statistically significant association or difference. Figure 1 shows the coproduction of ESBL + AmpC.

**Figure 2:**
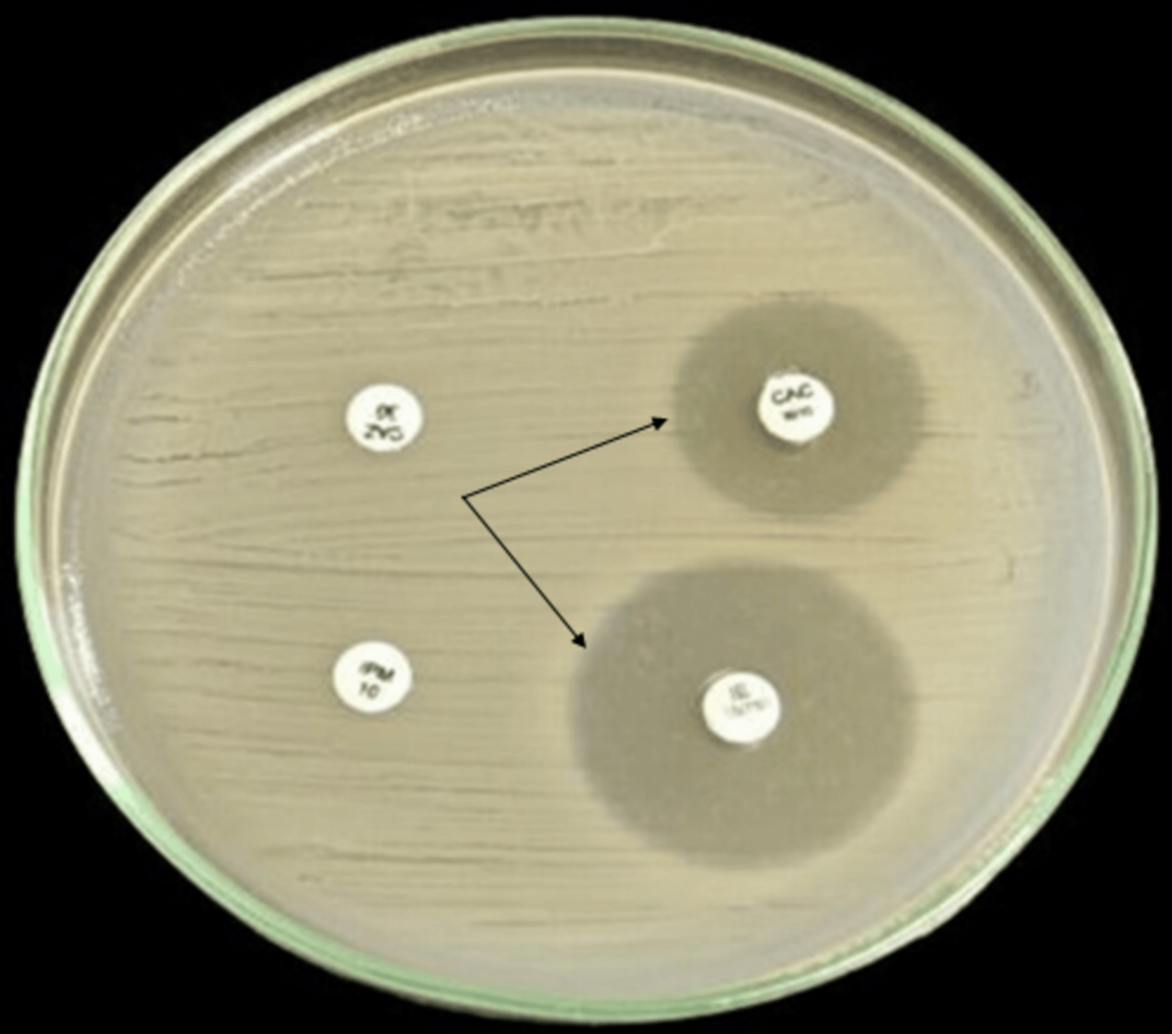
MBL + ESBL production MBL: metallo-β-lactamases; ESBL: extended spectrum of β-lactamases. The arrows indicate the coproduction of MBL and ESBL in the test isolate.

Figure 2 illustrates the coproduction of MBL + ESBL.

**Figure 3:**
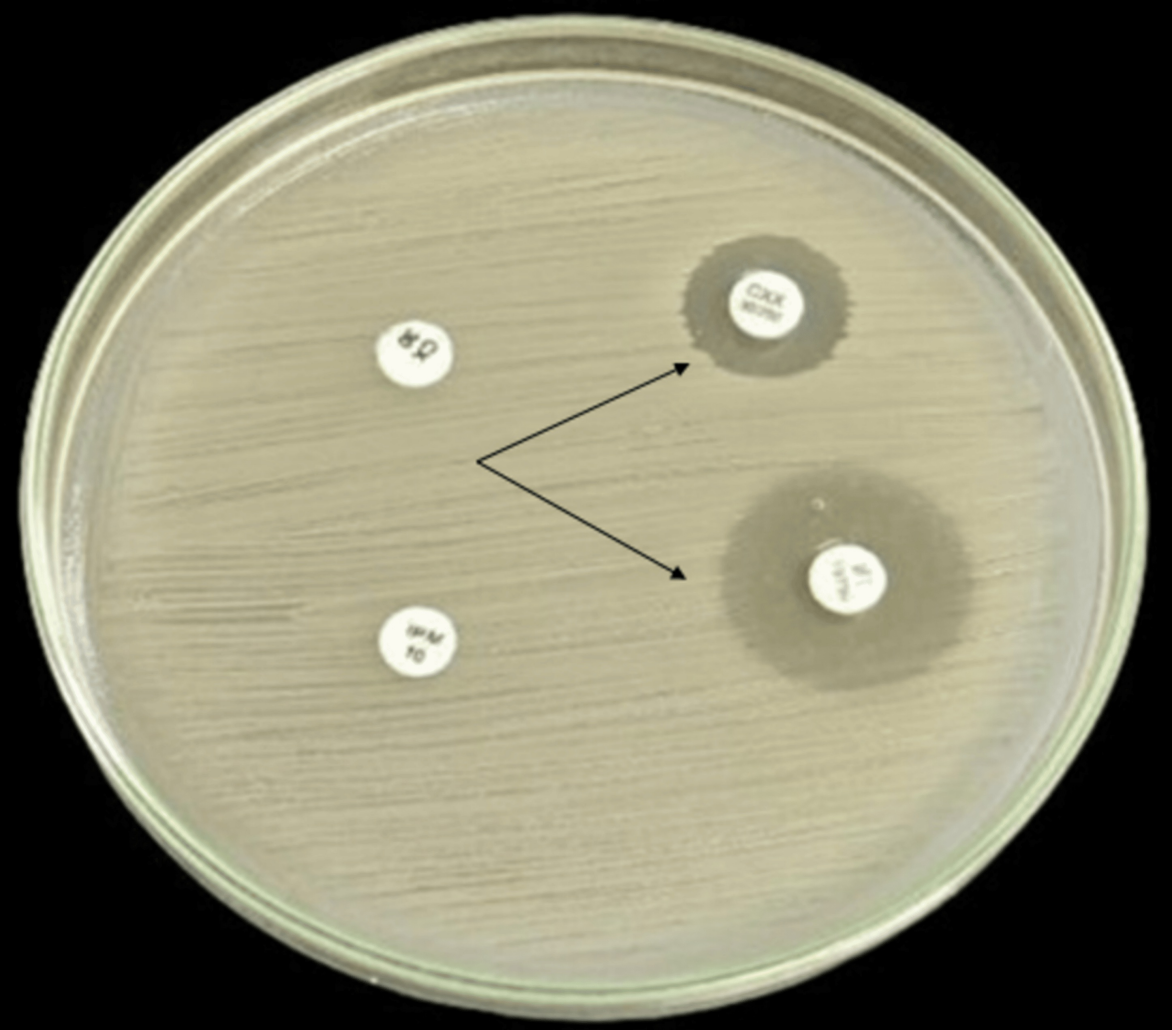
MBL + AmpC production MBL: metallo-β-lactamases; AmpC: AmpC β-lactamases. The arrows indicate the coproduction of MBL and AmpC β-lactamases in the test isolate.

Figure 3 shows the coproduction of MBL + AmpC.

**Figure 4:**
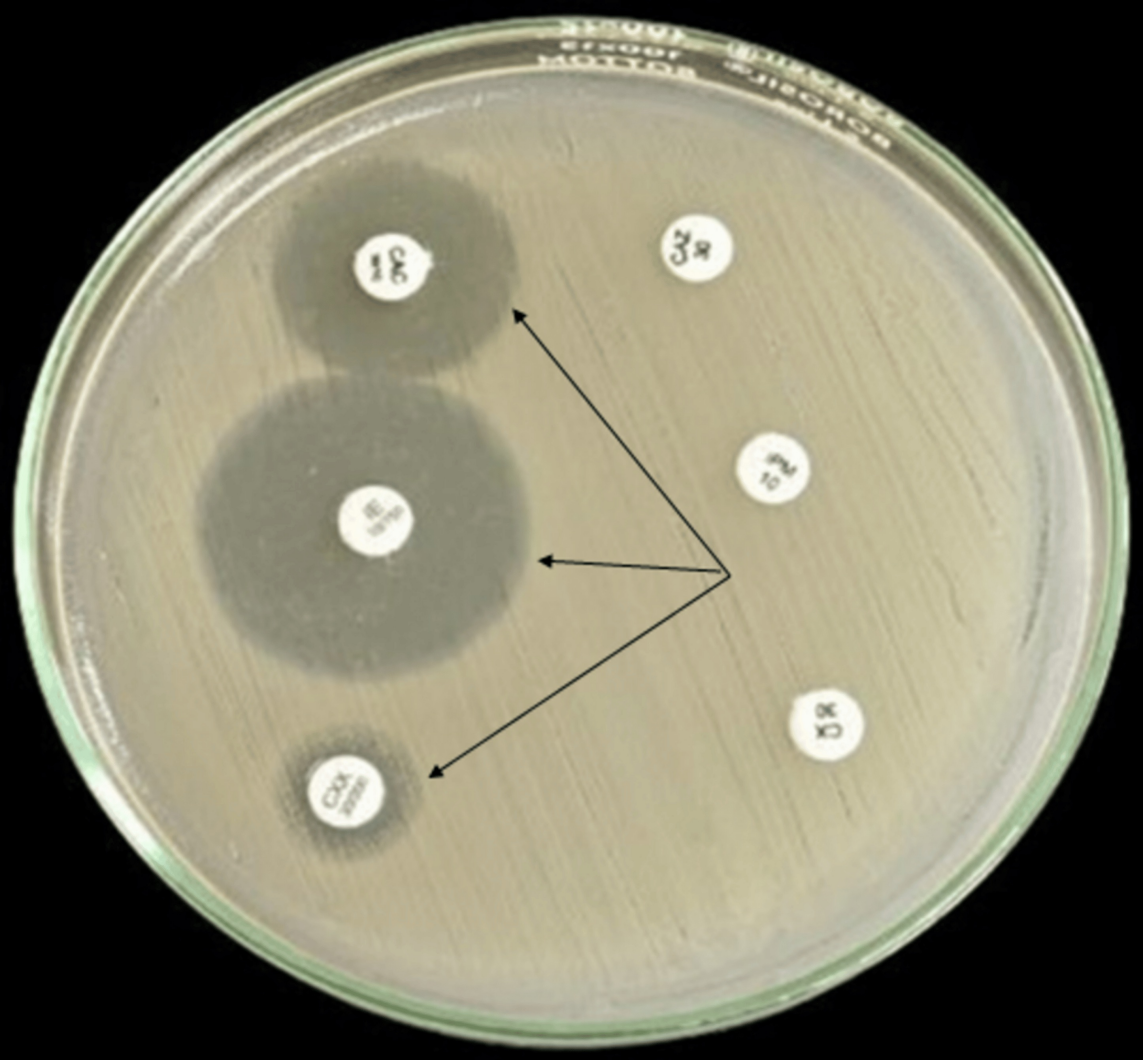
ESBL + MBL + AmpC production MBL: metallo-β-lactamases; ESBL: extended spectrum of β-lactamases; AmpC: AmpC β-lactamases. The arrows highlight the concurrent production of ESBL, MBL, and AmpC β-lactamases in the test isolate.

Figure 4 depicts the coproduction of ESBL + MBL + AmpC.

## Results

The data illustrated in Table 1 presents the distribution of Enterobacteriaceae isolates by age and sex. The highest frequency of isolates was identified in the 41-60 years age group, 60 (38.4%), followed by the 21-40 years group, 52 (33.3%). In terms of sex distribution, the highest number of male isolates was observed in the 41-60 years group, 42 (26.9%), followed by the 21-40 years group, 30 (19.2%), with the fewest in the 0-20 years group, 10 (6.4%). Among females, the 21-40 years group had the highest number of isolates, 22 (14.1%), followed by the 41-60 years group, 18 (11.5%), while the 0-20 years group had the fewest isolates, 8 (5.1%).

**Table 1:** Age, gender wise distribution of Enterobacteriaceae isolates N: the absolute number, %: the corresponding percent values.

Age	Male N (%)	Female N (%)	Total (N)	Total Percentage (%)
0-20	10 (6.4)	8 (5.1)	18	11.50
21-40	30 (19.2)	22 (14.1)	52	33.30
41-60	42 (26.9)	18 (11.5)	60	38.40
>60	14 (8.9)	12 (7.9)	26	16.80
Total	96 (61.4)	60 (38.6)	156	100

Of the 185 Gram-negative isolates as shown in Table 2, *Klebsiella pneumoniae* was the most prevalent organism, accounting for 90 (50.0%), followed by *Escherichia coli* with 60 (33.3%). The *Enterobacter cloacae* complex was isolated in 20 (11.1%), while *Proteus mirabilis* and *Citrobacter freundii* were isolated in 10 (5.6%) and 5 (2.8%) cases, respectively, making them the least frequent isolates.

**Table 2:** Enterobacteriaceae isolates with percentage N: the absolute number, %: the corresponding percent values.

Organism	No. of Isolates (N)	Percentage (%)
Escherichia coli	60	33.3%
Klebsiella pneumoniae	90	50.0%
*Enterobacter cloacae* complex	20	11.1%
Proteus mirabilis	10	5.6%
Citrobacter freundii	5	2.8%

Of a total of 145 MDR Enterobacteriaceae isolates, 63 (43.4%) elucidated the concurrent expression of two or three β-lactamases. In the study, 28 (19.3%) isolates demonstrated coexpression of MBL and ESBL (Table 3).

**Table 3:** Concurrent appearance of MBL and ESBL among Enterobacteriaceae isolates MBL: metallo-β-lactamases, ESBL: extended spectrum of β-lactamases, N: the absolute number, %: the corresponding percent values.

Organism	MBL + ESBL N (%)
Escherichia coli	10 (35.7)
Klebsiella pneumoniae	14 (50)
*Enterobacter cloacae *complex	3 (10.7)
Proteus mirabilis	1 (3.5)
Citrobacter freundii	0 (0)
Total	28 (19.3)

The coexpression of MBL and AmpC was observed in 13 (8.9%) isolates as indicated in Table 4.

**Table 4:** Concurrent appearance of MBL and AmpC among Enterobacteriaceae isolates MBL: metallo-β-lactamases, AmpC: AmpC β-lactamases, N: the absolute number, %: the corresponding percent values.

Organism	MBL + AmpC N (%)
Escherichia coli	4 (30.7)
Klebsiella pneumonia	7 (53.8)
*Enterobacter cloacae* complex	2 (15.3)
Proteus mirabilis	0 (0)
Citrobacter freundii	0 (0)
Total	13 (8.9)

Coexpression of ESBL and AmpC enzyme was detected in 16 (11%) isolates, as presented in Table 5.

**Table 5:** Concurrent appearance of ESBL and AmpC among Enterobacteriaceae isolates ESBL: extended spectrum of β-lactamases, AmpC: AmpC β-lactamases, N: the absolute number, %: the corresponding percent values.

Organism	ESBL + AmpC N (%)
Escherichia coli	6 (37.5)
Klebsiella pneumoniae	10 (62.5)
*Enterobacter cloacae* complex	0 (0)
Proteus mirabilis	0 (0)
Citrobacter freundii	0 (0)
Total	16 (11)

Simultaneous coproduction of MBL, ESBL, and AmpC enzyme was detected in 6 (4.1%) isolates, as demonstrated in Table 6, highlighting a concerning level of multi-enzyme resistance among the studied isolates.

**Table 6:** Concurrent appearance of MBL, ESBL, and AmpC among Enterobacteriaceae isolates MBL: metallo-β-lactamases, ESBL: extended spectrum of β-lactamases, AmpC: AmpC β-lactamases, N: the absolute number, %: the corresponding percent values.

Organism	MBL + ESBL + AmpC N (%)
Escherichia coli	2 (33.3)
Klebsiella pneumoniae	4 (66.67)
*Enterobacter cloacae* complex	0 (0)
Proteus mirabilis	0 (0)
Citrobacter freundii	0 (0)
Total	6 (4.1)

## Discussion

Antibiotics have revolutionized medicine since their discovery in the mid-20th century, significantly reducing morbidity and mortality. However, the rampant misuse of antibiotics has led to a global rise in antimicrobial resistance (AMR). Resistance patterns exhibit significant regional variation, with marked discrepancies between Asia and Western countries, driven by divergent antimicrobial policies and public health practices. In India, the true extent of AMR remains elusive due to the absence of a unified surveillance system, inconsistent investigation methods, and lack of standardization in laboratory practices [9].

As per current study, 96 (61.4%) were males with the majority and females were 60 (38.6%). Similar findings were reported by Negi et al. [10], stating that males were more affected as compared to the females. In Indian rural settings, there exists a notable disparity in outdoor mobility between men and women, with women generally exhibiting lower rates of outdoor activity. This behavioral pattern potentially contributes to a higher incidence of infections among men compared to women within these communities.

In the contemporary study, *Escherichia coli* accounted for 60 (33.3%), *Klebsiella pneumoniae* for 90 (50.0%), *Enterobacter cloacae* complex for 20 (11.1%), *Proteus mirabilis* for 10 (5.6%), and *Citrobacter freundii* for 5 (2.8%). The studies conducted by Thomas and Sarwat [11] showed highest occurrence of *Escherichia coli* (63.75%), whereas the highest isolation of *Klebsiella pneumoniae* was shown in our study with a percentage prevalence of 90 (50.0%).

Within the scope of this investigation, it was demonstrated that 28 (19.3%) of MDR Enterobacteriaceae coproduce both MBL and ESBL enzymes simultaneously. Our findings show higher prevalence compared to all other studies reviewed, with Salvia et al. (11.5%) being the exception, showing lower prevalence in comparison [12]. When examining isolates producing MBL and AmpC together, our results, 13 (8.9%), closely align with those of Jena et al. (9.73%) [13]. Moreover, our study shows the highest percentage of isolates producing both ESBL and AmpC, 16 (11%), compared to other studies. The prevalence of isolates capable of producing all three β-lactamases concurrently in our study was 6 (4.1%), slightly lower than that reported by Kolhapure et al., 194 (5.09%) [14].

Limitations of the study

This study was conducted at a single tertiary care center with a relatively limited sample size, which may restrict the generalizability of the findings to other healthcare settings and geographic regions. Additionally, only phenotypic methods were used for the detection of ESBL, AmpC, and MBL production; molecular characterization of resistance genes was not performed. Therefore, the specific genetic mechanisms underlying β-lactamase coproduction could not be identified. Further multicenter studies with larger sample sizes and molecular analysis are recommended to better understand the epidemiology and transmission of these resistant organisms.

## Conclusions

The study showcases the alarming prevalence of multidrug-resistant Enterobacteriaceae, particularly the concurrent expression of multiple β-lactamases, including MBL, ESBL, and AmpC. The coexistence of these enzymes within a single isolate, with 43.4% of multidrug-resistant strains producing multiple β-lactamases and 4.1% expressing all three, presents a significant challenge to treatment strategies. This underscores the urgent need for early detection of such resistance mechanisms. Therefore, to address this growing concern, it is essential to incorporate routine phenotypic testing for the detection of multiple β-lactamase production into standard microbiological diagnostic workflows. Collaborative efforts between clinicians and microbiologists are vital in fostering robust antimicrobial stewardship and mitigating the further spread of these resistant pathogens.
